# Spillover Benefits: Emphasizing Different Benefits of Environmental Behavior and Its Effects on Spillover

**DOI:** 10.3389/fpsyg.2018.02347

**Published:** 2018-12-13

**Authors:** Ellen Van Der Werff, Linda Steg

**Affiliations:** Environmental Psychology, University of Groningen, Groningen, Netherlands

**Keywords:** environmental benefits, monetary benefits, environmental behavior, environmental self-identity, spillover

## Abstract

To reduce environmental problems, people need to consistently engage in pro-environmental behaviors. Many environmentally friendly actions not only benefit the environment, but can also save money. Research suggests that emphasizing monetary benefits of pro-environmental behavior may hinder positive spillover to other pro-environmental behaviors. Yet, it is unclear why and under which circumstances this is the case. We propose that spillover effects depend on how emphasizing different types of benefits affects environmental self-identity, as a stronger environmental self-identity is more likely to lead to other pro-environmental actions. We hypothesize that emphasizing monetary benefits of pro-environmental behavior is less likely to strengthen environmental self-identity than emphasizing environmental benefits, and therefore not likely to lead to positive spillover. We tested our hypotheses in four experiments. In Study 1, we found that emphasizing the environmental benefits of pro-environmental behavior strengthened environmental self-identity, and resulted in positive spillover compared to not emphasizing any benefits or emphasizing monetary benefits. However, these results were not replicated in Study 2 that included a larger student sample. Yet, Study 3, including a large sample of the general population, showed that emphasizing monetary benefits weakens environmental self-identity and thereby leads to less spillover than emphasizing environmental benefits or not emphasizing any benefits. Similarly, Study 4 suggests that emphasizing monetary benefits may weaken environmental self-identity and decrease positive spillover compared to emphasizing environmental benefits or no benefits. Our findings suggest that environmental self-identity is not easily influenced by emphasizing different types of benefits of behavior, and consequently, spillover behavior is not easily promoted or inhibited. Yet, emphasizing monetary benefits may be a risk in some cases, as it may inhibit positive spillover.

## Introduction

To reduce environmental problems, people need to consistently engage in pro-environmental behavior (Intergovernmental Panel on Climate Change [IPCC], 2018). Policy makers aiming to promote pro-environmental behavior often do so by emphasizing the individual benefits of the behavior. For example, it is emphasized that saving energy also saves you money. Yet, emphasizing the monetary benefits of environmental behavior may be less effective in promoting the target behavior than emphasizing the environmental benefits (Bolderdijk et al., 2013; Schwartz et al., 2015). Monetary benefits of environmental behavior are often small, and may therefore not be perceived as worth the effort (Dogan et al., 2014). Importantly, emphasizing monetary benefits of pro-environmental behavior may not only hinder the adoption of the target behavior, but may also reduce the likelihood of spillover to other pro-environmental behaviors (Evans et al., 2013; Steinhorst et al., 2015; Steinhorst and Matthies, 2016). Spillover effects entail that the engagement in an initial pro-environmental behavior influences the likelihood of subsequent environmental actions (Thøgersen, 1999; Thøgersen and Ölander, 2003; Thøgersen and Crompton, 2009). An initial pro-environmental behavior can increase the likelihood of subsequent environmental behavior (i.e., positive spillover) or decrease the likelihood of following environmental behavior (i.e., negative spillover; Thøgersen and Crompton, 2009). To prevent negative spillover, and to promote positive spillover from initial pro-environmental behavior to subsequent pro-environmental actions, it is crucial to understand why and under which circumstances an initial behavior may lead to spillover when emphasizing different benefits.

A few studies suggest that emphasizing the monetary benefits of pro-environmental behavior hampers positive spillover to other pro-environmental actions. For example, when environmental benefits of car sharing were emphasized, people are afterward more likely to recycle compared to a control group, while there was no significant difference with the control group in recycling when monetary benefits of car sharing were emphasized (Evans et al., 2013). However, in this study people were presented with a scenario on car sharing, but it is not clear if they engaged in that behavior. Therefore, it is not yet clear how emphasizing benefits of behavior that people engaged in influences spillover effects. Similarly, emphasizing environmental benefits when providing electricity saving tips did promote other environmental behaviors compared to a control group, while emphasizing monetary benefits when providing those tips did not promote other environmental behaviors compared to a control group (Steinhorst et al., 2015; Steinhorst and Matthies, 2016). In this case, electricity saving tips were provided, and hence, no reference was made to whether people engaged in initial pro-environmental behavior. Furthermore, another study asked people to report their energy use and indicate how they would reduce their energy use by 5% (Spence et al., 2014). Next, participants received feedback that either emphasized environmental benefits of energy use or monetary benefits. The results showed that emphasizing environmental benefits of energy savings led to positive spillover compared to presenting financial benefits of energy savings; yet, in this study both experimental groups did not differ from the control condition in the extent to which spillover occurred. Again, it is not clear whether people actually engaged in the initial behavior, in this case energy saving behavior. Overall, these studies suggest that emphasizing the monetary benefits of pro-environmental behavior is less likely to result in positive spillover than emphasizing environmental benefits. However, the experimental conditions did not always significantly differ from the control condition. Therefore, it is important to study under which conditions spillover effects are most likely to occur. Furthermore, to provide more insight into how emphasizing benefits of pro-environmental behavior influences spillover, it is crucial to study the underlying process. We propose that spillover effects depend on the extent to which emphasizing different benefits strengthens the extent to which people realize they engaged in pro-environmental behavior and therefore see themselves as a person who engages in environmentally friendly behavior (i.e., when their environmental self-identity is strengthened) which in turn influences spillover effects. We propose that people are more likely to realize they engaged in pro-environmental behavior when environmental benefits of behavior are emphasized compared to when monetary benefits are emphasized.

Specifically, we reason that engagement in a pro-environmental behavior may particularly promote positive spillover to other environmental actions when the initial behaviors strengthen one’s environmental self-identity (Van der Werff et al., 2014b). Environmental self-identity is the extent to which people see themselves as a pro-environmental person (Van der Werff et al., 2013). Environmental self-identity reflects a person identity as defined by Stets and Burke (2000), and refers to how people see themselves. Environmental self-identity is partly stable, as it is influenced by someone’s values (Van der Werff et al., 2013; Gatersleben et al., 2014). However, how people see themselves is also malleable to some extent (Stets and Burke, 2000). For example, when initial environmentally friendly behavior signals that one is a pro-environmental person, environmental self-identity is likely to be strengthened. As people are motivated to be consistent and act in line with how they see themselves, a strong environmental self-identity in turn is likely to increase the likelihood of engagement in other pro-environmental behaviors (Stets and Burke, 2014). Indeed, a stronger environmental self-identity was associated with a range of pro-environmental actions, including self-reported behaviors such as energy conservation, reduction of waste, eco-shopping (Whitmarsh and O’Neill, 2010), recycling, refraining from flying to a holiday destination (Gatersleben et al., 2014), and with the likelihood of using green energy in the coming year, a stronger preference for sustainable products and actual use of paper in a more economical way (Van der Werff et al., 2013, 2014b). Hence, when people realize they engaged in environmentally friendly behavior, their environmental self-identity is likely to be strengthened, increasing the likelihood of positive spillover to other pro-environmental actions.

We propose that engagement in pro-environmental behavior for which monetary benefits have been emphasized reduces the extent to which the behavior signals that one is a pro-environmental person, thereby not strengthening environmental self-identity and not promoting spillover to other environmental behaviors. Specifically, people may be less likely to realize they engaged in pro-environmental behavior when the behavior is presented as having monetary benefits. Indeed, research suggests that when people engage in pro-environmental behavior for environmental reasons, environmental self-identity is strengthened and spillover to other pro-environmental behaviors is likely to occur (Peters et al., 2018). However, when people engage in pro-environmental behavior for other reasons, such as monetary reasons, their environmental self-identity is not strengthened and spillover to other pro-environmental behaviors is not likely (Peters et al., 2018), probably because in such cases, people are less likely to realize they engaged in pro-environmental behavior. Hence, we propose that for positive spillover to occur, it is critical that people realize they engaged in pro-environmental behavior, which is less likely to be the case when the monetary benefits of the particular behavior are emphasized.

In some cases, people may realize they engaged in environmentally friendly behavior even when the environmental benefits are not emphasized, for example when people believe the behavior has clear environmental benefits. For example, adopting solar panels or an electric vehicle may both be clearly seen as pro-environmental behaviors, even when environmental benefits are not emphasized. In such cases, emphasizing the environmental benefits of the behavior may have no or little added value above not stressing any benefit of the behavior, as people are likely to already realize they engaged in a pro-environmental behavior. When environmental benefits of behavior are very clear, engagement in such behavior is likely to strengthen environmental self-identity even when environmental benefits are not emphasized, making positive spillover to other pro-environmental behavior likely anyway. However, when the monetary benefits of such behaviors are emphasized, engagement in these behaviors may reduce the likelihood that people realize they engaged in pro-environmental behavior compared to not emphasizing any benefits, as they may instead see the behavior primarily as financially beneficial. Therefore, emphasizing the monetary benefits of a behavior that is clearly pro-environmental may weaken environmental self-identity and lead to less positive spillover compared to not emphasizing any benefits.

The current paper will test spillover effects following initial pro-environmental behavior for which monetary, environmental or no benefits are emphasized. Importantly, we will examine the underlying process through which emphasizing different benefits of behavior can influence spillover behavior. We expect that when people realize they engaged in a pro-environmental behavior, their environmental self-identity is more likely to be strengthened, making positive spillover to other environmental behavior more likely. Specifically, we expect environmental self-identity to be increased (rather than merely made salient) by reminding people of their past pro-environmental behavior. Research has shown that past environmental behavior is more likely to influence environmental self-identity when it concerns environmental behavior that they typically conduct, and not when it concerns environmental behavior which they hardly engage in (Van der Werff et al., 2014b). This suggests that environmental self-identity is not merely made salient by a reminder of environmental behavior, but that environmental self-identity increases when you realize you often engage in environmental behavior. In Study 1, we will focus on behavior that may not clearly be associated with environmental benefits, making it less likely that people realize they engaged in pro-environmental behavior. We expect that emphasizing the environmental benefits of these actions will strengthen environmental self-identity and spillover to other pro-environmental behavior compared to emphasizing monetary benefits or not emphasizing any benefits. To validate our findings, we will replicate Study 1 among a larger student sample in Study 2, and among a larger general population sample in Study 3. In Study 4, we focus on behavior that is clearly pro-environmental. When people anticipate engaging in such behavior, environmental self-identity may be strengthened and positive spillover may increase even when environmental benefits are not emphasized. We expect that emphasizing the monetary benefits of such behaviors may weaken environmental self-identity and reduce positive spillover compared to emphasizing environmental benefits or not emphasizing any benefits because emphasizing monetary benefits may make it less likely that people realize they engaged in a pro-environmental behavior.

## Study 1

### Methods

Data were collected via an online questionnaire. Participants were students of a university in the Netherlands participating in a course. Participants were invited via email to fill out the online study; they did not receive any compensation for it. In total, 39 participants filled out the questionnaire (*N* = 17 in the monetary condition, *N* = 9 for in environmental condition, *N* = 13 in the control condition). Age ranged from 18 to 29 (*M* = 21.3), 5 participants were male, 34 female.

#### Materials

We included a control question to check if participants carefully filled out the questionnaire. In the title of the question, we asked participants what their favorite sport is. However, in the explanation below the title we explained that this was a quality check and people should indicate what their favorite pet is, not their favorite sport. When participants mentioned a pet in their answer to this question they were included in the data analyses. Out of 39 participants, 35 answered this question by mentioning a pet (*N* = 15 in the monetary condition, *N* = 9 for in environmental condition, *N* = 11 in the control condition). We report the results based on all participants in the main text and the results based on the participants who answered the control question correct in a footnote.

We manipulated the type of benefit of respondents’ past pro-environmental behavior (following Cornelissen et al., 2008)^1^. Participants were presented with a list of eight behaviors that many people frequently engage in (switching off appliances; lowering the heating; going by bike instead of by car; returning returnable bottles; switching off lights when no-one is in the room; using energy efficient light bulbs; not eat meat every day; washing with a full load). Participants were asked to indicate to what extent each behavior applies to them (e.g., ‘I switch off electric appliances’) on a scale from 1 (totally disagree) to 7 (totally agree). As the behaviors are behaviors most people frequently engage in, the idea is that participants realize that they regularly engage in these behaviors. To emphasize the different benefits of the behaviors, the behaviors were either presented as environmental, monetary or neutral behaviors (e.g., ‘Please indicate to what extent the following statements on *environmental behavior/financial behavior/behavior* apply to you’). As expected, overall, participants frequently engaged in these behaviors (*M* = 5.51, *SD* = 0.74). There were no significant differences between the three conditions in the extent to which they agree with the statements [*F*(2,36) = 1.35, *p* = 0.27]; simple contrast further revealed that the environmental (*M* = 5.86, *SD* = 0.58), monetary (*M* = 5.43, *SD* = 0.50) and control condition (*M* = 5.38, *SD* = 1.01) all did not significantly differ (all *p*-values > 0.10).

#### Measures

The following three items were used to measure environmental self-identity: Acting environmentally friendly is an important part of who I am; I am the type of person who acts environmentally friendly; I see myself as an environmentally friendly person (Van der Werff et al., 2013). Respondents rated each item on a seven-point scale, ranging from totally disagree to totally agree. Cronbach’s alpha for this scale was 0.93 (*M* = 3.96, *SD* = 1.31).

To measure spillover, participants were asked to choose one out of two options of a product. One of the options was always the environmentally friendly and more expensive option, the other was the environmentally unfriendly and cheaper option. Participants indicated for five products: cookies, paper towel, deodorant, light bulbs, and cleaning products if they preferred the cheaper environmentally unfriendly option or the 10% more expensive environmentally friendly option. We counted the number of pro-environmental options participants chose out of the five options (*M* = 3.23, *SD* = 1.39).

### Results

**FIGURE 1:**
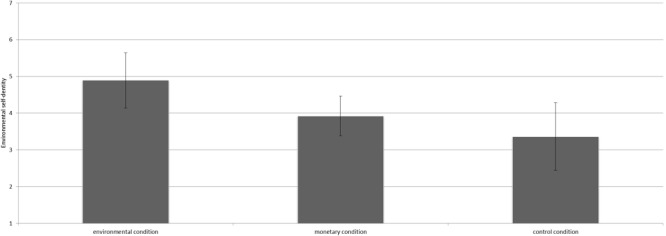
Mean scores on environmental self-identity for the three conditions including the 95% confidence interval.

We conducted analysis of variance (ANOVA) to test our hypotheses. The manipulation had a significant influence on environmental self-identity [*F*(2,36) = 4.27, *p* = 0.02, $\eta_{p}^{2}$ = 0.19^2^]. Contrast analyses revealed that participants in the environmental condition (*M* = 4.89, *SD* = 0.97) had a stronger environmental self-identity than those in the control condition [*M* = 3.36, *SD* = 1.52; *t*(36) = 2.92, *p* < 0.01, *d* = 1.20, see Figure 1]. Besides, participants in the environmental condition had a marginally significantly stronger environmental self-identity than those in the monetary condition [*M* = 3.96, *SD* = 1.10; *t*(36) = 1.94, *p* = 0.06, *d* = 0.90]. No differences in environmental self-identity were found between the monetary condition and the control condition [*t*(36) = 1.26, *p* = 0.22].

**FIGURE 2:**
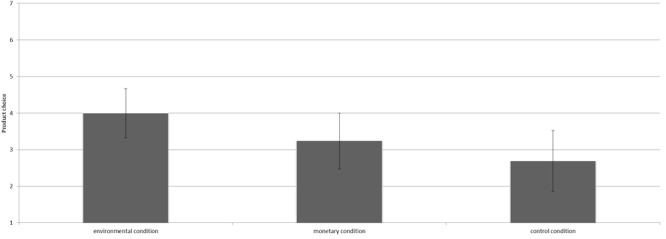
Mean number of environmentally friendly products chosen per condition including the 95% confidence interval.

The manipulation had a marginally significantly effect on product choice [*F*(2,36) = 2.57, *p* = 0.09, $\eta_{p}^{2}$ = 0.13^2^]. Contrast analyses revealed that participants in the environmental condition (*M* = 4.00, *SD* = 0.87) chose more pro-environmental products than those in the control condition [*M* = 2.69, *SD* = 1.38; *t*(36) = 2.27, *p* = 0.03, *d* = 1.14; see Figure 2]. Participants in the monetary condition (*M* = 3.24, *SD* = 1.48) chose less sustainable products than participants in the environmental condition, however, this difference was not statistically significant [*t*(36) = 1.39, *p* = 0.17]. The monetary and control condition did not differ significantly either [*t*(36) = 1.11, *p* = 0.28].

### Discussion

In Study 1, we found that emphasizing the environmental benefits of past behavior that are commonly adopted strengthens environmental self-identity and results in choosing more pro-environmental products compared to not emphasizing any benefits. Emphasizing monetary benefits of the same behaviors resulted in a marginally significantly weaker environmental self-identity compared to emphasizing environmental benefits. Environmental self-identity did not differ depending on whether monetary benefits or no benefits were emphasized. Emphasizing monetary benefits of past environmental behavior did not result in significantly less spillover behavior compared to emphasizing environmental benefits or no benefits. Our findings suggest that for pro-environmental behavior that is commonly adopted, emphasizing the environmental benefits does strengthen environmental self-identity and does lead to spillover behavior compared to a control group, while emphasizing monetary benefits does not strengthen environmental self-identity and does not promote positive spillover compared to a control group. One reason for not finding significant differences in spillover behavior between the environmental and monetary condition may be that the effects are too weak to detect in our sample. Therefore, Study 2 will include a larger student sample. Furthermore, in Study 1 the dependent variable was a choice between an environmentally friendly product that was more expensive and a cheaper environmentally unfriendly product. We argued that when people realize they engaged in pro-environmental behavior their environmental self-identity is strengthened and therefore they are more likely to choose the environmentally friendly products. However, it may be that when people realize they engaged in money saving behavior they may see themselves more as a person who saves money. Therefore, they may be more likely to choose the cheap products. This reasoning suggests that comparing people who realized they engaged in pro-environmental behavior to people who realized they engaged in money saving behavior may particularly lead to differences in environmental behavior that reflects a conflict between money and the environment. Therefore, in Study 2 we will also include a dependent variable that does not reflect a conflict between the environment and money.

## Study 2

### Methods

Data were collected via an online questionnaire. Participants were students of a university in the Netherlands participating in the study for credits. Power analysis showed that we needed 252 participants. In total, 366 participants filled out the questionnaire (*N* = 120 in the monetary condition, *N* = 125 for in environmental condition, *N* = 121 in the control condition). Age ranged from 17 to 38 (*M* = 19.9), 102 participants were male, 263 female, and 1 person indicated ‘other’ or preferred not to say.

#### Materials

We included the same control question as in Study 1 to check if participants carefully filled out the questionnaire. Out of 364 participants, 316 answered the question correct (*N* = 106 in the monetary condition, *N* = 106 for in environmental condition, *N* = 104 in the control condition). We report the results based on all participants in the main text and the results based on the participants who answered the control question correct in a footnote.

We manipulated the type of benefit of respondents’ past pro-environmental behavior in the same way as in Study 1. As expected, overall, participants regularly engaged in these behaviors (*M* = 5.45, *SD* = 0.81). There were no significant differences between the three conditions in the extent to which they engage in the behaviors [*F*(2,363) = 0.09, *p* = 0.92]. The control condition (*M* = 5.46, *SD* = 0.81), the environmental condition (*M* = 5.43, *SD* = 0.77), and the monetary condition (*M* = 5.47, *SD* = 0.87) did not significantly differ (all *p*-values > 0.10).

#### Measures

We used the same items as in Study 1 to measure environmental self-identity. Cronbach’s alpha for the scale was 0.89 (*M* = 4.77, *SD* = 1.29).

We used the same product choice task to measure spillover as in Study 1. On average participants chose 3.63 pro-environmental products out of five (*SD* = 1.24).

We measured the intention to engage in pro-environmental behaviors that are not associated with higher financial costs, with four items (I would sign a petition to protest against environmentally unfriendly policies; I support pro-environmental policies; I intend to recycle my waste; I intend to reduce my waste). Respondents rated each item on a seven-point scale, ranging from totally disagree to totally agree. Cronbach’s alpha for this scale was 0.79 (*M* = 5.54, *SD* = 1.15).

### Results

**FIGURE 3:**
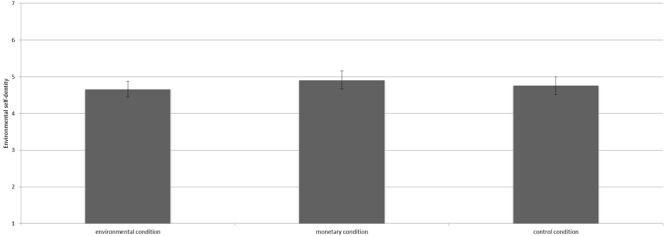
Means scores on environmental self-identity for the three conditions including the 95% confidence interval.

We conducted analysis of variance (ANOVA) to test our hypotheses. The manipulation did not significantly influence environmental self-identity [*F*(2,363) = 1.23, *p* = 0.29^3^]. Contrast analyses revealed that environmental self-identity did not differ for participants in the environmental condition (*M* = 4.66, *SD* = 1.19), the control condition (*M* = 4.76, *SD* = 1.33) and the monetary condition (*M* = 4.91, *SD* = 1.35; all *p*’s > 0.10, see Figure 3).

**FIGURE 4:**
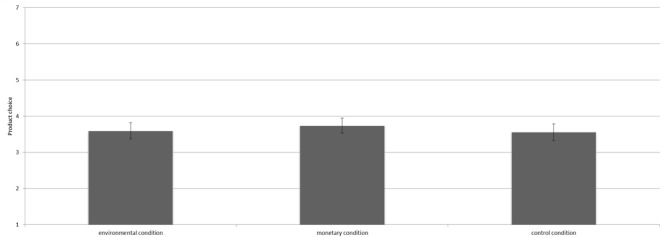
Mean number of environmentally friendly products chosen per condition including the 95% confidence interval.

The manipulation did not influence product choice [*F*(2,363) = 0.73, *p* = 0.48^3^]. Contrast analyses revealed that participants in the environmental condition (*M* = 3.59, *SD* = 1.26), the control condition (*M* = 3.56, *SD* = 1.28), and the monetary condition (*M* = 3.74, *SD* = 1.17) did not significantly differ in the number of pro-environmental products chosen (all *p*’s > 0.10, see Figure 4).

**FIGURE 5:**
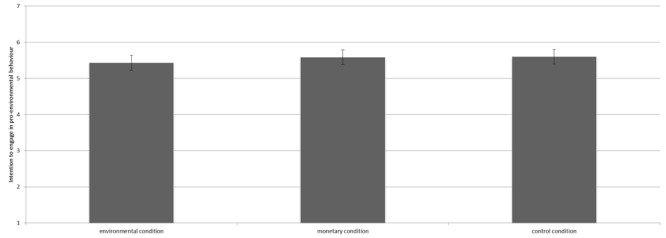
Mean intention to engage in pro-environmental behavior per condition including the 95% confidence interval.

The manipulation did not influence intention [*F*(2,363) = 0.84, *p* = 0.43^3^]. Contrast analyses revealed that participants in the environmental condition (*M* = 5.43, *SD* = 1.18), the control condition (*M* = 5.60, *SD* = 1.11), and the monetary condition (*M* = 5.59, *SD* = 1.14) did not significantly differ in intention to engage in pro-environmental actions (all *p*’s > 0.10, see Figure 5).

### Discussion

Study 2 aimed to replicate Study 1 among a larger student sample with sufficient power. Study 2 showed that emphasizing the environmental benefits of past pro-environmental behavior did not increase environmental self-identity compared to not emphasizing any benefits or compared to emphasizing the monetary benefits. Emphasizing the monetary benefits also did not reduce environmental self-identity compared to not emphasizing any benefits. Furthermore, emphasizing the environmental benefits of past environmental behavior did not increase the number of pro-environmental products chosen or the intention to engage in pro-environmental behavior compared to not emphasizing any benefits or compared to emphasizing the monetary benefits. Emphasizing the monetary benefits also did not influence product choice or intention compared to not emphasizing any benefits. Our hypotheses that emphasizing the environmental benefits of past behavior strengthens environmental self-identity and leads to spillover behavior are thus not confirmed. The findings suggest that environmental self-identity may be quite robust, and not easily changed by emphasizing different benefits of past pro-environmental actions. This is in line with research showing that environmental self-identity is partly stable because it is rooted in one’s values (Van der Werff et al., 2013; Gatersleben et al., 2014). Emphasizing the environmental or monetary benefits of the behavior may not easily influence environmental self-identity and spillover to other environmental actions. However, we tested our hypotheses among a rather specific sample, namely university students, mostly female. To test the validity of our findings further, we replicated the study again among a more general population sample.

## Study 3

### Methods

Data were collected via an online questionnaire. Participants were members of a Qualtrics panel in the Netherlands who received a small financial compensation for their participation. Power analysis showed that we needed 252 participants. In total, 307 participants filled out the questionnaire (*N* = 102 in the monetary condition, *N* = 102 for in environmental condition, *N* = 103 in the control condition). Age ranged from 18 to 81 (*M* = 50.6), 163 participants were male, 143 female, and 1 person indicated ‘other’ or preferred not to say.

#### Materials

We included two control questions to check if participants carefully filled out the questionnaire. The strict control question was the same question as in Studies 1 and 2. Out of 307 participants, 168 answered this control question correctly, namely by mentioning a pet (*N* = 56 in the monetary condition, *N* = 56 for in environmental condition, *N* = 56 in the control condition). We included a second control question as well, namely an item stating ‘I have paid attention so I will select “seven” on the scale.’ All participants selected seven for this scale. We report the results based on all participants in the main text and the results based on the participants who answered the control question correct in a footnote.

We manipulated the type of benefit of respondents’ past pro-environmental behavior in the same way as in Studies 1 and 2. As expected, overall, participants regularly engaged in these behaviors (*M* = 5.68, *SD* = 0.91). There were no significant differences between the three conditions in the extent to which they engaged in the behaviors [*F*(2,304) = 2.09, *p* = 0.13]. However, in the control condition (*M* = 5.77, *SD* = 0.92) participants indicated to engage in the behavior marginally significantly more than in the monetary condition [*M* = 5.54, *SD* = 1.01; *t*(304) = 1.89, *p* = 0.06]. Furthermore, in the environmental condition (*M* = 5.74, *SD* = 0.78) participants indicated to marginally significantly engage in the behavior more than in the monetary condition [*t*(304) = 1.63, *p* = 0.10]. The environmental and control condition did not significantly differ (*p* = 0.80). Importantly, in all conditions participants engaged in the behaviors frequently.

#### Measures

We used the same items as in Study 1 to measure environmental self-identity. Cronbach’s alpha for the scale was 0.92 (*M* = 5.16, *SD* = 1.29).

We used the same product choice task to measure spillover as in Study 1. On average participants chose 3.87 pro-environmental products out of five (*SD* = 1.28).

We used the same items to measure the intention to engage in pro-environmental behaviors as in Study 2. Cronbach’s alpha for this scale was 0.85 (*M* = 5.53, *SD* = 1.19).

### Results

**FIGURE 6:**
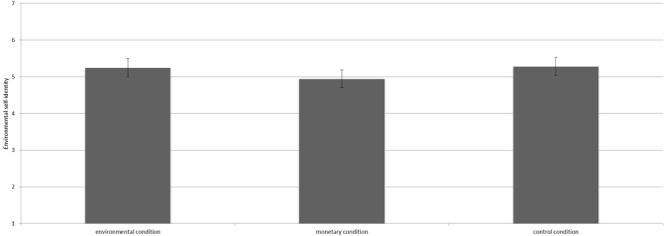
Means scores on environmental self-identity for the three conditions including the 95% confidence interval.

We conducted analysis of variance (ANOVA) to test our hypotheses. The manipulation did not significantly influence environmental self-identity [*F*(2,304) = 2.13, *p* = 0.12^4^]. Contrast analyses revealed that environmental self-identity of participants in the environmental condition (*M* = 5.25, *SD* = 1.31) did not differ from the control condition (*M* = 5.28, *SD* = 1.26; *p* = 0.84). However, the monetary condition (*M* = 4.94, *SD* = 1.28) scored marginally significantly weaker on environmental self-identity than the control condition [*t*(304) = 1.88, *p* = 0.06, see Figure 6]. Furthermore, participants in the monetary condition reported a marginally significantly weaker environmental self-identity than participants in the environmental condition [*t*(304) = 1.67, *p* = 0.10].

**FIGURE 7:**
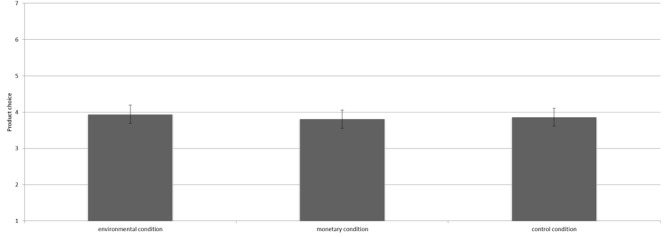
Mean number of environmentally friendly products chosen per condition including the 95% confidence interval.

The manipulation did not influence product choice [*F*(2,304) = 0.25, *p* = 0.78^5^]. Contrast analyses revealed that participants in the environmental condition (*M* = 3.94, *SD* = 1.17), the control condition (*M* = 3.86, *SD* = 1.32), and the monetary condition (*M* = 3.81, *SD* = 1.36) did not significantly differ in the number of products they chose (all *p*’s > 0.10, see Figure 7).

**FIGURE 8:**
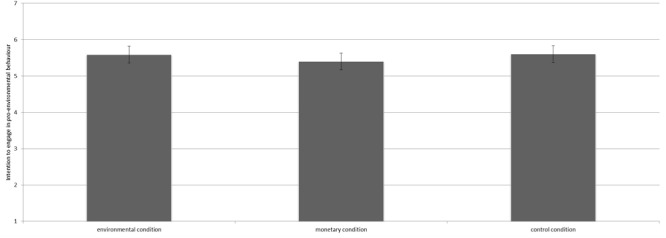
Mean intention to engage in pro-environmental behavior per condition including the 95% confidence interval.

The manipulation did not influence intention to engage in pro-environmental behavior [*F*(2,304) = 0.89, *p* = 0.41^5^]. Contrast analyses revealed that intentions did not significantly differ across participants in the environmental condition (*M* = 5.59, *SD* = 1.10), the control condition (*M* = 5.60, *SD* = 1.11), and the monetary condition (*M* = 5.40, *SD* = 1.34; all *p*’s > 0.10, see Figure 8).

As the sample size in this study was relatively large and the effects of the manipulation on environmental self-identity are marginally significant, we additionally tested if environmental self-identity mediates the relationship between the manipulation and product choice and intention to act pro-environmentally, respectively. As reported above, environmental self-identity only marginally differed between the monetary condition versus the control condition as well as the environmental condition. Therefore, we computed a dummy variable comparing the monetary condition to both other conditions. We used Hayes’ macro to test if the dummy variable influenced product choice and intention via environmental self-identity (Hayes et al., 2010).

The results showed that the dummy variable influenced product choice via environmental self-identity (*a* × *b* = -0.14). The 95% confidence interval ranged from -0.30 to -0.01^5^. As the confidence interval did not include 0, the mediation effect was significant. Emphasizing the monetary benefits weakened environmental self-identity compared to not emphasizing benefits or emphasizing environmental benefits (*a* = -0.32, *p* = 0.04). Next, environmental self-identity was positively related to pro-environmental product choice (*b* = 0.44, *p* < 0.001). The direct effect of the dummy variables on product choice remained not significant when environmental self-identity was also included in the analysis (*c’* = 0.05, *p* = 0.72). Therefore, we found indirect-only mediation (Zhao et al., 2010).

The dummy variable influenced intention to act pro-environmentally via environmental self-identity as well (*a* × *b* = -0.22). The 95% confidence interval ranged from -0.45 to -0.01^5^. As the confidence interval did not include 0, the mediation effect was significant. Environmental self-identity was positively related to the intention to engage in pro-environmental behavior (*b* = 0.70, *p* < 0.001). The direct effect of the dummy variable on intention remained not significant when environmental self-identity was included as well (*c’* = 0.05, *p* = 0.75). Therefore, again, we found indirect-only mediation (Zhao et al., 2010).

### Discussion

Study 3 aimed to replicate Studies 1 and 2, again with sufficient power, but this time we tested our hypotheses among a more general population sample. The results show that emphasizing environmental benefits of past environmental behavior does not strengthen environmental self-identity compared to the control group. However, emphasizing the monetary benefits of past behavior marginally significantly weakens environmental self-identity compared to the not emphasizing any benefits and compared to emphasizing the environmental benefits. We did not find any direct effects of the manipulation on spillover to pro-environmental product choice or intention to engage in pro-environmental behavior. However, the results of the mediation analyses show that emphasizing the monetary benefits of behavior weakens environmental self-identity and thereby reduces both types of positive spillover behavior. We found that a weakened environmental self-identity resulted in less pro-environmental behavior that reflects a conflict between money and the environment. However, importantly, a weakened environmental self-identity also resulted in a weaker intention to engage in pro-environmental behavior that does not cost money. These findings suggest that our results are not only explained because emphasizing the monetary benefits makes people focus more on the financial benefits and therefore makes financially beneficial behavior more likely. It suggests that when monetary benefits are emphasized, environmental self-identity is weaker, making it less likely that people engage in other pro-environmental behaviors, also when these behaviors are not financially costly.

However, again, our findings suggest that people’s environmental self-identity rather robust as it is not easily changed by emphasizing different types of benefits of past behavior. This is in line with research showing that environmental self-identity is partly stable because it is rooted in one’s values. Therefore, emphasizing the environmental or financial benefits of the behavior is not likely to easily promote positive spillover to other pro-environmental behaviors. Interestingly, it seems that if any effect occurs emphasizing monetary benefits may be risky, as this may weaken environmental self-identity to some extent.

## Study 4

We reasoned in the introduction that emphasizing the monetary benefits may particularly weaken environmental self-identity and lead to less positive spillover to other pro-environmental behavior when a behavior is clearly pro-environmental. When behavior is clearly pro-environmental, people are likely to be well aware of the environmental benefits. Emphasizing monetary benefits of such behavior may merely weaken the extent to which people perceive its environmental benefits and therefore weaken environmental self-identity and positive spillover to other pro-environmental behaviors. Therefore, Study 4 aimed to test if emphasizing the monetary benefits of a clearly pro-environmental behavior results in a weaker environmental self-identity and reduces positive spillover to other pro-environmental actions compared to not emphasizing any benefits of the initial behavior or emphasizing the environmental benefits of the behavior.

### Methods

Data were collected via an online questionnaire study. Participants were students in a course who could participate on a voluntary basis and did not receive any compensation for it. Participants were invited to participate in the study via email. In total, 91 participants filled out the questionnaire (*N* = 30 in the monetary condition, *N* = 30 for in environmental condition, *N* = 31 in the control condition). Age ranged from 19 to 28 (*M* = 21.9), 32 participants were male, and 59 female.

#### Materials

We included the same strict control question as in Studies 1, 2, and 3 to check if participants carefully filled out the questionnaire. Out of 91 participants, 71 answered this question by mentioning a pet (*N* = 21 in the monetary condition, *N* = 26 for in environmental condition, *N* = 24 in the control condition). We report the results based on all participants in the main text and the results based on the participants who answered the control question correct in a footnote.

We manipulated past pro-environmental behavior via a scenario. Participants were randomly assigned to one of three scenarios: the monetary condition, environmental condition and control condition^6^. Participants were asked to imagine that they just bought an electric vehicle. We asked participants to imagine they spent a lot of time figuring out which car to buy and that few others would buy an electric vehicle, thereby strengthening the extent to which purchasing an electric vehicle says something about a person and thereby strengthening its influence on environmental self-identity (see Van der Werff et al., 2014a). The adoption of an electric vehicle in the scenario was either presented as a pro-environmental behavior, a financially beneficial behavior or no emphasis was included:

‘Imagine that you work at a company and need a car to get to work every day. You bought an electric car. You spent a lot of time figuring out which electric car *was most environmentally friendly to buy/was financially most attractive to buy/to buy*. You chose a car that was *very environmentally friendly/the best financial investment*. Only few people buy an electric car.’

#### Measures

The same items as in the previous studies were used to measure environmental self-identity. Cronbach’s alpha for this scale was 0.90 (*M* = 4.59, *SD* = 1.11).

Similarly to the product choice task in Studies 1, 2, and 3 participants, were asked to choose one out of two options of a product to measure spillover effects. One of the options was always an environmentally friendly option that was 10% more expensive, while the other was an environmentally unfriendly but cheaper option. For example, participants were asked to choose between a pair of socks of 3 Euros that was produced unsustainably or a pair of socks of 3.30 Euros that was produced sustainably. In this study, participants indicated for eight products, namely jeans, milk, a laptop, a pen, a writing pad, a bike, a pair of socks and a mobile phone which option they preferred. We counted the number of pro-environmental options participants chose out of eight options (*M* = 4.40, *SD* = 2.21).

### Results

**FIGURE 9:**
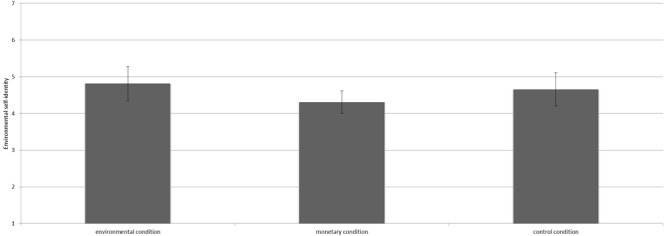
Means scores on environmental self-identity for the three conditions including the 95% confidence interval.

We conducted analysis of variance (ANOVA) to test our hypotheses. The manipulation did not have a significant effect on environmental self-identity [*F*(2,85) = 1.61, *p* = 0.21, $\eta_{p}^{2}$ = 0.04^7^]. However, contrast analyses revealed that participants in the monetary condition (*M* = 4.31, *SD* = 0.82) reported a marginally significantly weaker environmental self-identity than participants in the environmental condition [*M* = 4.82, *SD* = 1.21; *t*(85) = 1.75, *p* = 0.08, *d* = 0.49]. Environmental self-identity did not significantly differ between the monetary condition and the control condition [*M* = 4.66, *SD* = 1.21; *t*(85) = -1.21, *p* = 0.23, see Figure 9]. As expected, no differences in environmental self-identity were found between participants in the environmental condition and participants in the control condition [*t*(85) = 0.56, *p* = 0.58].

**FIGURE 10:**
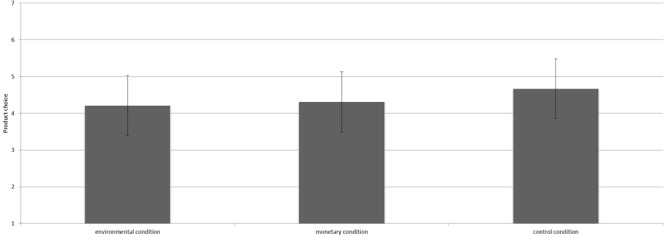
Mean number of environmentally friendly products chosen per condition including the 95% confidence interval.

The manipulation did not have a significant effect on product choice [*F*(2,85) = 0.35, *p* = 0.71, $\eta_{p}^{2}$ = 0.01^8^]. Contrast analyses revealed that participants in the environmental condition (*M* = 4.21, *SD* = 1.99), the control condition (*M* = 4.67, *SD* = 2.51), and the monetary condition (*M* = 4.31, *SD* = 2.14) did not significantly differ in the number of pro-environmental products chosen (all *p*’s > 0.10, see Figure 10).

We again tested if environmental self-identity mediated the relationship between the manipulation and spillover behavior. As reported above, environmental self-identity only marginally differed between the monetary condition versus the environmental condition. Therefore, we computed a dummy variable comparing the monetary condition to the other conditions. We again used Hayes’ macro to test if the dummy variable influenced product choice via environmental self-identity.

The results showed that the dummy variable influenced product choice via environmental self-identity (*a* × *b* = -0.48). The 95% confidence interval ranged from -1.06 to -0.01^9^. As the confidence interval did not include 0, the mediation effect was significant. Emphasizing the monetary benefits marginally significantly weakened environmental self-identity compared to not emphasizing benefits or emphasizing environmental benefits (*a* = -0.42, *p* = 0.09). Next, environmental self-identity was positively related to pro-environmental product choice (*b* = 1.13, *p* < 0.001). The direct effect of the dummy variables on product choice remained not significant when environmental self-identity was also included in the analysis (*c’* = 0.35, *p* = 0.42). Therefore, we found indirect-only mediation (Zhao et al., 2010).

### Discussion

In Study 4, we tested in a scenario study the influence of emphasizing monetary benefits of buying an electric car, which is likely to be a clearly pro-environmental behavior, on environmental self-identity and spillover behavior. Our findings again suggest that environmental self-identity is quite robust and not easily changed by emphasizing different benefits of the behavior. However, we found that emphasizing the monetary benefits of buying an electric vehicle marginally significantly weakens environmental self-identity compared to emphasizing the environmental benefits. We did not find effects of emphasizing different benefits on pro-environmental product choice. However, we did find that emphasizing the monetary benefits marginally significantly weakens environmental self-identity and thereby weakens spillover behavior as environmental self-identity mediated the relationship between the manipulation and spillover behavior. When we only included the participants who answered the control question correct emphasizing monetary benefits lead to a weaker environmental self-identity compared to not emphasizing any benefits and compared to emphasizing environmental benefits. Among the participants who answered the control question correct, emphasizing monetary benefits also reduced the number of pro-environmental products chosen compared to not emphasizing any benefits. However, emphasizing the environmental benefits also reduced the number of pro-environmental products chosen compared to not emphasizing any benefits. Overall, these findings particularly suggest that emphasizing the monetary benefits of clearly pro-environmental behavior is risky, because environmental self-identity is weakened compared to emphasizing environmental benefits thereby reducing spillover effects.

## General Discussion

We aimed to test the influence of emphasizing different types of benefits of pro-environmental behavior on spillover to other pro-environmental behaviors. Research has shown that past pro-environmental actions can promote spillover to other pro-environmental behaviors by strengthening one’s environmental self-identity. We proposed that emphasizing the monetary benefits of behavior may hamper the extent to which initial pro-environmental behavior strengthens environmental self-identity and promotes spillover to other pro-environmental behaviors, as doing so may decrease the likelihood that people realize they engaged in pro-environmental behavior.

Our results partly support our reasoning. Notably, results of Study 1 show that emphasizing the environmental benefits of environmental behavior that people commonly engage in does strengthen environmental self-identity and promotes spillover to other pro-environmental behaviors compared to not emphasizing any benefits. In contrast, emphasizing the monetary benefits of common pro-environmental actions does not strengthen environmental self-identity and does not result in stronger positive spillover effects compared to not emphasizing any benefits. Yet, in Study 2 we found that emphasizing environmental benefits does not increase environmental self-identity and spillover to other pro-environmental behavior. Furthermore, emphasizing monetary benefits did not weaken environmental self-identity and did not lead to less spillover to other pro-environmental behavior. In Study 3, we found that emphasizing monetary benefits somewhat weakens environmental self-identity compared to not emphasizing any benefits or emphasizing environmental benefits and thereby reduces spillover to other pro-environmental behaviors. However, we did not find direct effects of emphasizing different types of benefits on spillover behavior. Additionally, Study 4 shows that emphasizing monetary benefits of behavior with clear environmental benefits may somewhat weaken environmental self-identity and thereby reduce positive spillover to other pro-environmental behavior compared to emphasizing environmental benefits. However, we again did not find direct effects on spillover behavior. When we only included those who answered the control question correctly, Study 4 showed that emphasizing monetary benefits of behavior with clear environmental benefits weakens environmental self-identity compared to not emphasizing any benefits and compared to emphasizing environmental benefits. Furthermore, emphasizing monetary benefits reduced spillover compared to not emphasizing any benefits. However, emphasizing environmental benefits also reduced spillover behavior compared to not emphasizing any benefits for those participants.

Our research extends previous research in three ways. First, we tested the effects of an initial pro-environmental action on other pro-environmental behavior. We either reminded people of their past environmental behavior or presented them with a scenario in which they were asked to imagine they adopted a pro-environmental behavior. In earlier studies people did not actually engage in the initial pro-environmental action (Evans et al., 2013; Spence et al., 2014; Steinhorst et al., 2015; Steinhorst and Matthies, 2016). We sometimes found spillover effects. Yet, often we did not find direct spillover effects. To better understand under which circumstances spillover effects occur it is important that future research on spillover includes an initial environmental behavior. Initial environmental behavior can be included through a reminder of past behavior or a scenario. However, importantly, future research is also needed to test spillover behavior following actual environmental behavior.

Second, we studied the process underlying possible spillover effects. Our findings suggest that spillover effects depend on the extent to which environmental self-identity is strengthened. Notably, initial behaviors are more likely to encourage engagement in other types of pro-environmental behavior when the initial behavior strengthens individuals’ environmental self-identity.

Third, we studied the conditions under which spillover effects occur. Importantly, our findings suggest that environmental self-identity is quite robust, and not easily changed by emphasizing the monetary or environmental benefits of behavior. Therefore, spillover behavior is not likely to be easily promoted. This may be explained by the finding the environmental self-identity also has a stable component, as it is influenced by and rooted in the values that people endorse, particularly biospheric values. As a consequence, environmental self-identity is likely to be somewhat robust (Van der Werff et al., 2013; Gatersleben et al., 2014), and may only be changed when behavior clearly signals that you are a pro-environmental person (Van der Werff et al., 2014a). This is more likely when people realize they engaged in many pro-environmental behaviors or when the behavior is difficult and unique (Van der Werff et al., 2014a). Future research is needed to test under which circumstances emphasizing benefits of behavior is likely to influence environmental self-identity and thereby spillover behavior.

However, when people’s environmental self-identity can be changed our results particularly suggest that emphasizing monetary benefits can be risky. Notably, our findings indicate that initial pro-environmental behavior may weaken environmental self-identity and thereby not lead to positive spillover when monetary benefits of pro-environmental behavior were emphasized compared to environmental benefits. This was the case for behavior that people frequently engage in as well as behavior that is clearly pro-environmental. Environmental self-identity was in some cases also somewhat weakened when monetary benefits were emphasized compared to not emphasizing any benefits. Our findings suggest that it may easier to weaken environmental self-identity by emphasizing monetary benefits than to strengthen environmental self-identity by emphasizing environmental benefits. Future research could test whether it is indeed easier to weaken environmental self-identity than to strengthen environmental self-identity and why this may be the case.

Future research is needed to examine if spillover effects indeed depend on the extent to which people realize they engaged in pro-environmental behavior. Furthermore, it could be tested if similar processes play a role when it concerns other benefits of pro-environmental behavior, as well as when it concerns other samples. For example, research suggests that emphasizing health benefits of pro-environmental behavior may also prevent positive spillover to other environmental actions (Carrico et al., 2017). Furthermore, people may engage in pro-environmental behavior for status reasons (Griskevicius et al., 2010). Emphasizing status benefits may also weaken the extent to which people realize they engaged in pro-environmental behavior. Future research is needed to test if emphasizing other benefits such as health or status benefits also hampers the extent to which pro-environmental behavior strengthens environmental self-identity as people may not realize they engaged in pro-environmental behavior. Furthermore, future research could test if our findings can be replicated among different samples as well. In Study 3, we included a general population sample to validate our findings. However, our participants were still all Western participants. Future research is needed to test if our findings also apply to other samples in other cultures.

Future research could examine the role of other potential mediators that can explain why spillover effects occur, such as self-efficacy. Indeed, research suggests that environmental behavior may lead to spillover to other environmental actions by strengthening self-efficacy (Lauren et al., 2016). When people engage in a pro-environmental action this may strengthen the extent to which they think they can engage in pro-environmental behavior thereby increasing the likelihood of other pro-environmental behaviors. Future research could also test the mediating role of environmental self-identity and self-efficacy, as identity and self-efficacy may be related (Brenner et al., 2018). That is, the more one sees oneself as a pro-environmental person the more one may think that one is capable of engaging in pro-environmental behavior. Future research should study the relationships between identity and self-efficacy.

Our results suggest that environmental self-identity is rather robust and spillover behavior is not easily changed by emphasizing different types benefits of behavior. However, in some cases emphasizing the environmental or monetary benefits of past pro-environmental behavior may influence environmental self-identity and subsequently the likelihood of positive spillover. Interestingly, we did not only find some support for spillover to behavior that implied a choice between saving money and the environment we also found some support for spillover to pro-environmental behavior that does not cost money. This suggests that our findings are not explained because emphasizing monetary benefits makes people see themselves more as a person who saves money and therefore engage in behavior that saves money. Our findings suggest that when people realize they engaged in pro-environmental behavior, their environmental self-identity is strengthened and therefore they are more likely to choose the environmentally friendly product. However, more research is needed to test if emphasizing the environmental or monetary benefits of behavior influences pro-environmental behavior that does not reflect a conflict between the environment and money. Furthermore, future research could test the influence on behavior that benefits the environment and saves money such as saving energy.

Future research could test to which behaviors environmental self-identity is most strongly related, and thereby to which environmental behaviors spillover effects are most likely. Additionally, future research is needed to test spillover effects to actual pro-environmental behavior. We tested spillover effects on intention to engage in pro-environmental behavior and on hypothetical choices, namely the preference for pro-environmental but more expensive products. The question remains whether similar results are found when the behavior is more difficult, and when people actually need to pay the additional costs. In line with the ABC-theory, environmental self-identity may be most strongly related to behavior that is somewhat difficult (Stern, 2000). When environmental behavior is very easy, almost everyone may engage in the behavior, therefore individual factors such as environmental self-identity are not or hardly related to the behavior. When the behavior is very difficult, hardly anyone engages in the behavior, therefore individual factors such as environmental self-identity may also hardly or not be related to the behavior. Future research could test the extent to which actual behaviors and environmental self-identity are related, and the extent to which an initial pro-environmental behavior is likely to spillover to actual pro-environmental actions via one’s environmental self-identity. Future research could also examine whether effects depend on the extent to which the spillover behaviors are visible. There is some initial evidence to suggest that environmental identity is more strongly related to behaviors that can be observed by others than to behaviors that are not visible for others (Brick et al., 2017). However, this study focused on an environmental social identity, not environmental self-identity. Visibility may be particularly relevant for social identity, when people are motivated to act in line with what their group values. Visibility of the behavior may be less relevant for environmental *self*-identity, as people with a strong environmental self-identity are motivated to act in line with how they see themselves, not how others see them. Future research is needed to test if the visibility of the environmental behavior influences the relationship between environmental self-identity and visible behavior and thereby whether an initial pro-environmental behavior is more likely to spillover to visible environmental behaviors than to less visible behaviors.

We included control questions in the questionnaire to test if participants carefully read the questions. In Studies 1 and 2, only few participants answered the question incorrectly, and the results did not differ depending on whether we included those who answered the control question incorrectly or not. However, in Studies 3 and 4, many participants answered the control question incorrectly. In Study 3, the results remained similar when only those who answered the control question correctly were included. However, this time environmental self-identity only differed between the monetary and the control condition, no longer between the monetary and the environmental condition. In Study 4, those in the environmental condition still reported a stronger environmental self-identity than those in the monetary condition, but the monetary condition also differed significantly from the control condition. Moreover, the direct effect of the manipulation on product choice became significant. Participants in the monetary condition chose less pro-environmental products than those in the control condition. However, those in the environmental condition also chose less products than those in the control condition. Our findings suggest that in some cases it may be useful to include a control question. When the sample consists of students who participate in the study to receive credits or consists of a panel that receives a financial compensation for participating the participants may be less likely to read the questions carefully. In such cases it may be useful to include a control question to ensure that participants read the questions and answered the questions seriously.

In contrast to our expectations, we found that emphasizing environmental benefits of behavior that has clear environmental benefits may reduce positive spillover slightly compared to not emphasizing any benefits. However, we only found this in Study 4, when only those participants were included who answered the control question correct and the difference was only marginally significant. However, these findings may hint to a reactance effect. For an environmental behavior with clear environmental benefits, people may realize that they engaged in a pro-environmental behavior without emphasizing the environmental benefits. When it is emphasized that the behavior is environmentally friendly reactance may occur as people may feel manipulated by this emphasis. As a consequence, environmental self-identity may not be strengthened and people may not be willing to engage in other pro-environmental behaviors as well. Future research is needed to test if this finding can be replicated. If this is indeed the case, it should be tested if reactance can indeed explain our findings in Study 4. Furthermore, it could be that particularly people who do not care about the environment show this reactance effect. When you do not care about the environment, but the environmental benefits of a clearly environmental behavior are emphasized, this may particularly lead to reactance. Therefore, future research could measure other variables such as biospheric values to test if the influence of reminding people of environmental behavior on environmental self-identity and spillover behavior depends on factors such as the strength of one’s values.

Our findings may have important implications for studies testing incentives to promote environmental behavior, in which monetary benefits are not merely emphasized but actually provided in order to promote pro-environmental behavior. Research on spillover following incentives for pro-environmental behavior is mixed. Some studies suggest that incentivized environmental behavior may lead to positive spillover to other behaviors. For example, a monetary compensation for the purchase of sustainable products increased the purchase of these products compared to not receiving monetary compensation. Subsequently, the group purchasing more sustainable products was also more likely to engage in other pro-environmental behaviors (Lanzini and Thøgersen, 2014). Other studies suggest that incentivized pro-environmental behavior is less likely to lead to positive spillover compared to pro-environmental behavior that was not incentivized (Poortinga et al., 2013; Thomas et al., 2016). More specifically, providing people with a financial incentive to reduce the use of plastic bags seemed to effectively reduce the targeted behavior. Yet, in countries where people did not receive the monetary incentive for the initial behavior, positive spillover effects were stronger compared to countries where they did receive a monetary incentive. Also, emphasizing environmental benefits of electricity savings did lead to positive spillover to reducing waste in China. However, waste was not reduced when people received monetary incentives to reduce their energy, suggesting that incentives may reduce positive spillover effects (Xu et al., 2018). Our findings may provide insight into these mixed findings. Based on our findings, we propose that whether or not incentivized behavior promotes positive spillover to other environmental behaviors depends on the extent to which the incentivized behavior strengthens one’s environmental self-identity. More specifically, we propose that when people realize they engaged in a pro-environmental action, their environmental self-identity is strengthened and positive spillover is likely to occur. People may still realize they engaged in a pro-environmental action after engaging in incentivized environmental behavior. For example, compensating people for pro-environmental behavior as was done in the study by Lanzini and Thøgersen (2014) may still have increased the extent to which people realize they engaged in a behavior with environmental benefits, and see themselves as a pro-environmental person. After all, people did purchase pro-environmental products while they overall did not receive money as it was merely a compensation for the extra costs of the environmentally friendly products. However, when people receive strong financial incentives for pro-environmental behavior or when the emphasis is on the financial incentive, they may be less likely to realize they engaged in a behavior with environmental benefits. In that case, they may mainly see the behavior as providing monetary benefits not environmental benefits making positive spillover to other pro-environmental behaviors less likely. Future research is needed to test if spillover effects following financial incentives depends on the extent to which the incentivized behavior makes people realize they engaged in a pro-environmental action. Furthermore, future research is needed to test how incentives can be designed to ensure that the behavior increases environmental self-identity thereby promoting spillover to other environmental behaviors.

Our findings have important practical implications for policy aimed to promote spillover effects. To promote positive spillover to many environmental behaviors it is crucial that people realize they engaged in environmentally friendly behavior. Yet, at least in some cases, emphasizing the monetary benefits of environmental behaviors may be risky as it can weaken the extent to which people realize they engaged in pro-environmental action, making it less likely that environmental self-identity will be strengthened and weakening positive spillover. To promote positive spillover, it seems important that policy makers and practitioners instead emphasize the environmental benefits, as this makes it more likely that engaging in such behavior strengthens environmental self-identity and promotes positive spillover. For example, on recycling bins or cycling lanes messages could be added that emphasize the environmental benefits of this behavior. That way, people are more likely to realize they engage in pro-environmental actions thereby strengthening environmental self-identity making spillover to a range of pro-environmental behaviors more likely. However, as explained above, environmental self-identity is rather robust, so the appeals need to be sufficiently strong. Yet, being rather robust suggests that once environmental self-identity is strengthened it is likely to lead to long term pro-environmental behavior as it is not easily weakened.

## Ethics Statement

This study was carried out in accordance with the recommendations of “the National Code of Ethics for Research in the Social and Behavioural Sciences involving Human Participants as formulated by the National Ethics Council for Social and Behavioural Sciences, Ethical Committee Psychology of the University of Groningen” with informed consent from all subjects. All subjects gave informed consent in accordance with the Declaration of Helsinki. The protocol was approved by the “Ethical Committee Psychology of the University of Groningen.”

## Author Contributions

EVDW and LS designed the studies. EVDW collected and analyzed the data and drafted the article. LS engaged in several rounds of critical revision of the article.

## Conflict of Interest Statement

The authors declare that the research was conducted in the absence of any commercial or financial relationships that could be construed as a potential conflict of interest.

## Footnotes

1 The study also included a fourth condition in which participants were presented with a list of eight behaviors that have no clear monetary or environmental implications (e.g., playing games studying). However, as these were not environmental behaviors this condition is not relevant for the current study.

2 We ran the same analysis including only the participants who answered the control question correctly. The results are similar.

3 We ran the same analysis only including the participants who answered the control question correctly. The results are similar.

4 We ran the same analysis including the participants who answered the strict control question correctly. The results are similar. The control and monetary condition still differed [*t*(165) = 2.01, *p* = 0.05]. However, the monetary condition no longer differed significantly from the environmental condition [*t*(165) = 1.18, *p* = 0.24].

5 When only the participants who answered the strict control question correctly were included the results are similar.

6 Similar to Study 1, this study also included a fourth condition in which participants were not presented with a scenario. In this condition, an initial pro-environmental behavior was not manipulated therefore spillover cannot be tested. Therefore, this condition is not included in the current study.

7 When only the participants who answered the control question correct were included in the analyses the overall effect was significant [*F*(2,68) = 3.30, *p* = 0.04, $\eta_{p}^{2}$ = 0.09]. Those in the monetary condition (*M* = 4.03, *SD* = 0.77) reported a significantly weaker environmental self-identity than participants in the environmental condition [*M* = 4.64, *SD* = 1.15; *t*(68) = 1.97, *p* = 0.05, *d* = 0.62] and the control condition [*M* = 4.81, *SD* = 1.16; *t*(68) = -2.45, *p* < 0.05, *d* = 0.79]. The environmental and control condition remained not significantly different.

8 When only the participants who answered the control question correct were included in the analyses the overall effect was significant [*F*(2,68) = 2.82, *p* = 0.07, $\eta_{p}^{2}$ = 0.08]. Participants in the monetary condition (*M* = 3.67, *SD* = 2.03) chose significantly less sustainable products than participants in the control condition [*M* = 5.04, *SD* = 2.20; *t*(68) = -2.24, *p* < 0.05, *d* = 0.65]. Participants in the monetary condition did not differ from participants in the environmental condition [*M* = 4.00, *SD* = 1.94; *t*(68) = 0.55, *p* = 0.58]. Participants in the environmental condition chose marginally significantly less sustainable products than participants in the control condition [*t*(68) = -1.79, *p* = 0.08, *d* = 2.41].

9 When only the participants who answered the strict control question correctly were included the results are similar.
